# Subjective memory complaints, mood and MCI: a follow-up study

**DOI:** 10.1080/13607863.2015.1081150

**Published:** 2015-09-02

**Authors:** Jennifer A. Yates, Linda Clare, Robert T. Woods, MRC CFAS

**Affiliations:** ^a^ Division of Rehabilitation & Ageing, School of Medicine, University of Nottingham, Nottingham, UK; ^b^ Department of Psychology, University of Exeter, Exeter, UK; ^c^ Dementia Services Development Centre, Bangor University, Gwynedd, UK

**Keywords:** depression, anxiety, cognitive impairment

## Abstract

**Objectives:** Subjective memory complaints (SMC) are common in older people and previous research has shown an association with mood problems, such as depression and anxiety. SMC form part of the criteria for many definitions of mild cognitive impairment (MCI), but there is controversy over whether they should be included as they may be related more strongly to mood than to objective cognitive impairment. This study aims to clarify the relationship between mood and SMC in people with MCI.

**Method:** This paper reports an analysis of data from the Medical Research Council Cognitive Function and Ageing study. Structured interviews were conducted with community-dwelling older people to assess a range of aspects of cognitive functioning and mood. Data from two time points approximately 24 months apart were used in this analysis. At baseline, participants without dementia or severe cognitive impairment were categorised into three groups according to cognitive status. Mood was investigated by assessing symptoms of anxiety and depression which were defined using a diagnostic algorithm. Associations were tested using logistic regression and chi square analyses.

**Results:** A clear association was shown between SMC and mood, both cross-sectionally and over time. The relationship between our two competing definitions of MCI suggested that mood problems were more strongly related to the presence of SMC than objective cognitive impairment.

**Conclusion:** SMC may be a function of anxiety and depression rather than being related to objective cognitive function. This questions whether SMC should be included in definitions of MCI.

## Abbreviations

ADL: Activities of daily living
CAMCOG: Cambridge Cognitive Evaluation
CAMDEX: Cambridge Mental Disorders of the Elderly Examination
GMS-AGECAT: Geriatric Mental State Automated Geriatric Examination for Computer Assisted Taxonomy
MCI: Mild cognitive impairment
MCIW: Mild cognitive impairment without subjective memory complaints
MMSE: Mini-Mental State Exam
MRC-CFAS: Medical Research Council Cognitive Function and Ageing Study
OCIND: Other cognitive impairment, no dementia
OR: Odds ratio
SMC: Subjective memory complaints

## Introduction

Subjective memory complaints (SMC) are reports of problems with, or changes in, memory and are common in older people (Balash et al., 2013; Dux et al., 2008). Assessments of SMC range from brief questions concerning individuals' perceived memory function or how memory changes may have affected activities of daily living (ADL) (Cook & Marsiske, 2006) to more in-depth questionnaires, such as the Memory Functioning Questionnaire (Gilewski, Zelinski, & Schaie, 1990) or the Metamemory in Adulthood Questionnaire (Dixon, Hultsch, & Hertzog, 1988). SMC are associated with a lower quality of life in older people (Iliffe & Pealing, 2010; Mol, van Boxtel, Willems, & Jolles, 2006).

Petersen et al. (2001) make a connection between SMC and mild cognitive impairment (MCI) – a concept developed to describe a transitional phase between age-appropriate cognitive functioning and pathological decline (Matthews, Stephan, McKeith, Bond, & Brayne, 2008). However, this is controversial with other researchers suggesting that SMC are not an essential criterion for MCI and may lack both specificity and sensitivity as a diagnostic criterion (Lenehan, Klekociuk, & Summers, 2012). Accordingly, SMC are included as a criterion in only 10 of the 19 MCI definitions identified by the Medical Research Council Cognitive Function and Ageing Study (MRC-CFAS) study (Matthews et al., 2008; Stephan, Brayne, McKeith, Bond, & Matthews, 2008) alongside the requirement for an objective impairment in memory or other cognitive domains, such as language, absence of dementia, intact general cognitive functioning and intact ADL (Petersen, 2004; Petersen et al., 2001; Petersen et al., 1999).

Including SMC as a criterion for classification of MCI reduces the prevalence estimates of MCI (Matthews et al., 2008), in that as many as 62% of individuals who experience cognitive decline do not report SMC (Iliffe & Pealing, 2010). Possible reasons for such a discrepancy may include individual variations in adapting to cognitive change, where some individuals may not perceive such changes as significant or requiring action. However, progression to dementia in one study was predicted better by the presence of memory complaints than by global cognitive impairment without dementia or by domain-specific cognitive impairments. Palmer, Backman, Winblad, and Fratiglioni (2003) found that 51% of future dementia cases in a sample drawn from a population-based study had memory complaints.

SMC have also been related to depression and anxiety (McDougall, Becker, & Arheart, 2006). Depression or anxiety may influence the expression of SMC. Depression is positively associated with SMC (Minett, Da Silva, Ortiz, & Bertolucci, 2008; Zandi, 2004) and may enhance negative attributions (Roberts, Clare, & Woods, 2009) so that individuals may experience a distorted subjective appraisal of their memory function in the presence of depressive symptoms. SMC without objective impairment may be a manifestation of depressive symptoms (Balash et al., 2013). An increase in anxiety has also been associated with an increase in SMC despite no decrease in objective memory performance (Dux et al., 2008). Anxiety was found to be higher in individuals with SMC who have lower Mini Mental State Examination scores (MMSE) (Balash et al., 2013; Folstein, Folstein, & McHugh, 1975). Symptoms of depression and anxiety are increased in individuals who have been classified as having MCI (Barnes, Alexopoulos, Lopez, Williamson, & Yaffe, 2006; Bhalla et al., 2009; Chan, Kasper, Black, & Rabins, 2003; Geda et al., 2006; Yates, Clare, & Woods, 2013), potentially indicating a risk factor for the development of MCI, a reaction to the onset of cognitive decline or a circular relationship involving both possibilities. Depression may also form part of a prodromal phase of dementia, which would justify a triple relationship between depression, SMC and cognitive decline (Minett et al., 2008).

This study aimed to clarify the relationship between mood and SMC in people with MCI, in order to add to the discussion over whether SMC should be included as a criterion in the MCI definition. Previous research has established the association between SMC and mood, but this study has contributed to the field by investigating the role of SMC through their absence, which is a novel approach, not previously applied to the CFAS data-set.

This study aimed to answer the following research questions.

(1)  Are people with MCI more likely to have symptoms of anxiety or depression than people with normal cognitive functioning?
(2)  Are people with SMC more likely to report symptoms of anxiety or depression than people without SMC?
(3)  Does anxiety or depression at baseline predict the presence of SMC two years later?
(4)  Is anxiety or depression at baseline associated with a change in cognitive status over two years?
(5)  Will a change in cognitive status over two years predict the presence of anxiety or depression at the end of the two-year period?

## Methods

### Design

Mood and the presence of SMC were assessed longitudinally in a sample of community-dwelling older people who were participating in the MRC-CFAS. MRC-CFAS is a longitudinal population-based study involving participants drawn from five centres which represent rural and urban areas of England and Wales, investigating changes that affect people as they age. Participants were initially screened regarding their general health and day-to-day activities. Twenty per cent of the sample, including all those with apparent cognitive impairment on a screening measure and a proportion of other participants (randomly selected, stratified for age, geographical location and gender) went on to complete a more detailed assessment. Initial screening took place during 1991–1993. Participants were assessed again after approximately 24 months, between 1993 and 1995. Ethical approval was granted by University and NHS Ethics Committees and participants provided fully informed consent before taking part. This paper presents the analysis of baseline and follow-up data. The analyses reported in this paper were conducted in 2014.

### Participants

Individuals over 65 years and living in the Gwynedd, Cambridge, Nottinghamshire, Newcastle and Oxford areas of the United Kingdom were randomly sampled from 1990 to 1991. Fuller details are reported elsewhere (Brayne, McCracken, Matthews, & MRC-CFAS, 2006). The participants investigated in this study consisted of the 20% subsample who took part in the detailed assessment, drawn from the larger baseline sample. Data from the first assessment and two-year follow up interviews were used in this analysis. Participants were excluded from analyses if they had objective cognitive impairment beyond the criteria for MCI [dementia *n* = 587; other cognitive impairment, no dementia (OCIND) *n* = 234] or impaired ADLs (*n* = 475) resulting in 1344 participants included at the first assessment and 896 participants included at the two-year follow-up.

### Definition of subjective memory complaints

SMC were indicated by a self-report of memory problems by the participant. This was assessed using two questions from the baseline screening interview, ‘Have you ever had difficulty with your memory?’ and ‘Have you tended to forget things recently?’, and one question from the combined screen and interview ‘Have you had any difficulty with your memory?’ A positive answer to any of the above questions resulted in a participant being categorised as having an SMC at baseline, with SMC being a dichotomous category. SMC at follow-up were identified by a positive answer to either of two questions from the assessment: ‘Have you had any difficulty with your memory?’ or ‘Have you tended to forget things recently?’ Again, SMC was a dichotomous category.

### Assessment of mood

Anxiety and depression were defined by the Geriatric Mental State Automated Geriatric Examination for Computer Assisted Taxonomy (GMS-AGECAT) (Copeland, Dewey, & Griffiths-Jones, 1986) algorithm, which was calculated from questions asked during the MRC-CFAS interview (http://www.cfas.ac.uk). Traditionally, a GMS-AGECAT score of two indicates a subcase and a score of three or above indicates a case of anxiety or depression. In this study, participants with scores of two and above for anxiety or depression were considered to be a case in order to account for borderline and milder symptoms.

### Classification of cognitive status

**Figure 1.**
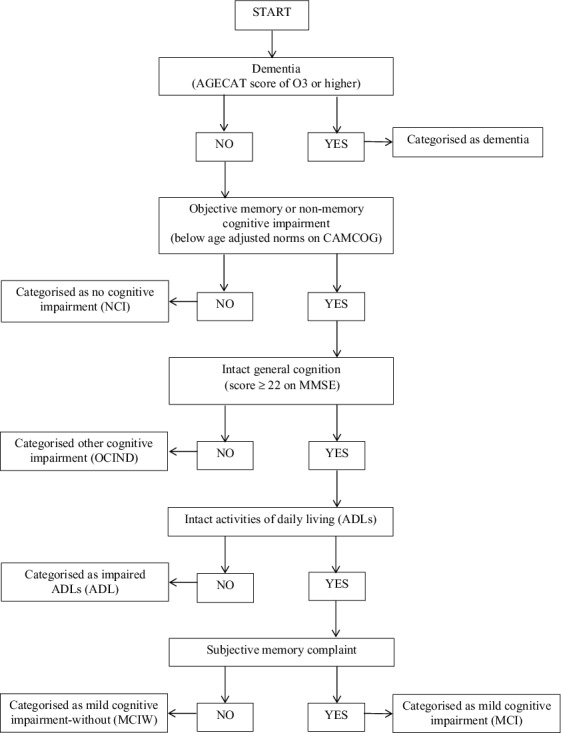
Algorithm used to allocate participants to each cognitive status category.

Several cognitive status categories are referred to throughout this study and were used to classify individuals at various points along the continuum from normal ageing to dementia. An algorithm was used to allocate participants to each cognitive status category (Figure 1). MCI was a broad definition using criteria similar to Petersen (Petersen et al., 1999), where participants categorised as MCI had an objective cognitive impairment (defined as a CAMDEX Cambridge Cognitive Examination score falling one standard deviation below age-adjusted norms), no dementia (shown as an AGECAT score of below O3), intact ADLs (defined using questions asked within the MRC-CFAS interview), intact general cognitive function (indicated by a score of 22 or higher on the MMSE) and SMC. The criterion of intact general cognition was included in order to maintain similarity in the MCI definition used with other studies that have used data from CFAS I.

A further cognitive status category was used at baseline which included participants who met all the criteria for MCI, except that they did not report SMC: these were described as MCI-without (MCIW; see Figure 1).

At follow-up, participants could be categorised as having MCI, MCIW, or dementia, or could be classified in two further categories (Figure 1). Some participants were classified as having other cognitive impairment, no dementia (OCIND) which comprised individuals who had general cognitive decline (defined by an MMSE score as lower than 22), but did not meet criteria for dementia and had intact ADLs. This group included participants with and without SMC. Participants could also be classified in the ADL category, which included people who had general cognitive decline (defined by an MMSE score of less than 22), impairments in ADLs but did not meet criteria for dementia. Again, this category included participants with and without SMC.

### Statistical analyses

Analyses were conducted using SPSS 20.0. Differences between participants with and without SMC were described for both time points. Logistic regression was used to calculate odds ratios (OR) for symptoms of anxiety or depression at baseline and follow-up according to cognitive status and presence of SMC. Pearson's chi-squared test was used to calculate changes in the presence of SMC and changes in cognitive status in the presence of symptoms of anxiety and depression over two years.

## Results

**Figure 2.**
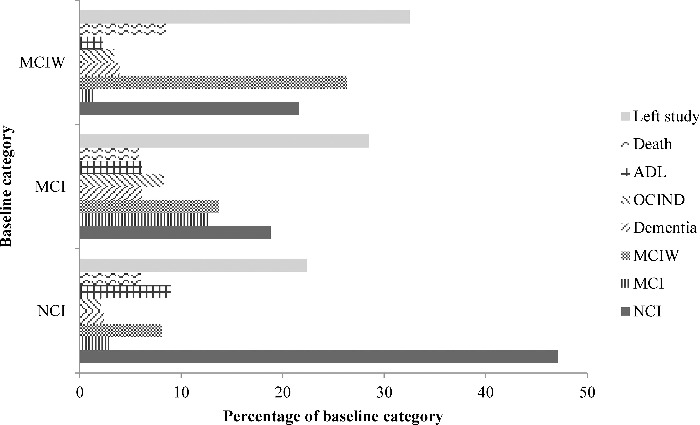
Changes in cognitive status between baseline and follow-up. Baseline category is shown on the *Y*-axis and each bar shows the percentage of participants who have moved to each category at follow-up.

Table 1 summarises the characteristics of the study sample at baseline and follow-up according to whether SMC were reported. At baseline, 557 participants from a total of 1344 (41.4%) participants reported SMC. There did not appear to be differences between those with SMC and those without in terms of age, MMSE scores, years in full time education or gender. Table 2 summarises the cognitive status of the participants according to whether or not SMC were reported. Due to the definitions used to create the cognitive classifications, all participants classified as having MCI reported SMC, and none of the participants classified as having MCIW reported SMC. The cognitive status of some participants changed between baseline and follow-up and this is shown in Figure 2. According to the definitions for cognitive impairment used in this study, 49.5% of participants were classified as having MCI or MCIW at baseline and 28.6% of participants were classified as having MCI or MCIW at follow-up.

**Table 1.** Sample characteristics for participants with and without subjective memory complaints at baseline and follow-up.

	Baseline		Follow-up	
	No SMC	SMC	No SMC	SMC
Age mean (sd)	73.69 (6.15)	74.56 (6.50)	75.41 (6.17)	76.68 (6.62)
MMSE mean (sd)	25.13 (3.69)	24.86 (3.49)	25.40 (3.47)	25.07 (3.13)
Female N (%)	501 (63.7)	360 (64.6)	433 (65.7)	151 (63.7)
Years in FT education mean (sd)	9.86 (2.13)	10.03 (2.23)	9.98 (2.12)	10.12 (2.32)
Without depression (%)	629 (79.9)	370 (66.4)	546 (82.9)	141 (59.5)
With depression (%)	158 (20.1)	187 (33.6)	113 (17.1)	96 (40.5)
Without anxiety (%)	766 (97.3)	514 (92.3)	651 (98.8)	214 (90.3)
With anxiety (%)	21 (2.7)	43 (7.7)	8 (1.2)	23 (9.7)
Total (%)	787 (58.6)	557 (41.4)	659 (73.5)	237 (26.5)

**Table 2.** Cognitive status according to SMC at baseline.

	Baseline		Follow-up	
N(%)	No SMC	SMC	No SMC	SMC
NCI	399 (50.7)	280 (50.3)	360 (54.6)	94 (39.7)
MCI	0 (0.0)	277 (20.6)	0 (0.0)	61 (6.8)
MCIW	388 (49.3)	0 (0.0)	195 (29.6)	0 (0.0)
OCIND			35 (5.3)	15 (6.3)
ADL			47 (7.1)	39 (16.5)
Dementia			22 (3.3)	26 (11.0)
Total	787 (58.6)	557 (44.4)	659 (73.5)	237 (26.5)

NCI = not cognitively impaired.

MCI = participants show objective cognitive impairment, intact general cognition, intact ADLs, no dementia, report of SMC.

MCIW represents participants who would otherwise be classified as MCI but do not report an SMC.

OCIND = other cognitive impairment, no dementia where participants indicate general cognitive decline but have intact ADLs and do not meet criteria for dementia.

ADL = participants who show general cognitive decline and impaired ADLs but do not meet criteria for dementia.

Dementia = participants who have been classified as having dementia.

Logistic regressions were conducted for each cognitive status group at baseline to investigate the odds of the presence of symptoms of anxiety or depression and results are shown in Table 3. The odds of having symptoms of anxiety or depression at baseline were significantly increased in participants classified as having MCI but were significantly decreased in those classified as MCIW relative to participants without cognitive impairment. The odds of having symptoms of anxiety at follow-up were not significantly increased or decreased for any cognitive status at baseline; however, the odds of having symptoms of depression at follow-up were increased in participants classified as having MCI relative to participants without cognitive impairment.

**Table 3.** Logistic regressions to show odds of anxiety or depression dependent on cognitive status.

Baseline cognitive status			
Anxiety at baseline	OR	CI	P
NCI	0.75	0.45–1.25	.268
MCI	3.22	1.93–5.38	.000**
MCIW	0.34	0.16–0.72	.005*
Depression at baseline			
NCI	1.04	0.82–1.33	.736
MCI	1.71	1.28–2.27	.000**
MCIW	0.58	0.44–0.78	.000**
Anxiety at follow-up			
NCI	0.90	0.44–1.85	.775
MCI	1.64	0.74–3.62	.224
MCIW	0.69	0.28–1.71	.423
Depression at follow-up			
NCI	0.82	0.60–1.11	.198
MCI	1.87	1.30–2.67	.001*
MCIW	0.72	0.50–1.05	.089
Follow-up cognitive status			
Anxiety at follow-up			
NCI	0.52	0.25–1.10	.086
MCI	1.49	0.44–5.05	.521
MCIW	0.24	0.06–1.02	.052
Dementia	1.23	0.29–5.31	.783
OCIND	0.00	0.00	.997
ADL	7.84	3.69–16.63	.000**
Depression at follow-up			
NCI	0.54	0.39–0.74	.000**
MCI	2.28	1.33–3.91	.003*
MCIW	0.72	0.48–1.07	.105
Dementia	1.10	0.56–2.16	.778
OCIND	1.59	0.86–2.95	.139
ADL	2.96	1.87–4.68	.000**

NCI = not cognitively impaired.

MCI = participants show objective cognitive impairment, intact general cognition, intact ADLs, no dementia, report of SMC

MCIW represents participants who would otherwise be classified as MCI but do not report an SMC.

OCIND = other cognitive impairment, no dementia where participants indicate general cognitive decline but have intact ADLs and do not meet criteria for dementia.

ADL = participants who show general cognitive decline and impaired ADLs but do not meet criteria for dementia.

Dementia = participants who have been classified as having dementia.

*Significant at *p* = .05 level, **Significant at *p* < .001 level.

Logistic regressions were conducted to determine the odds of symptoms of anxiety or depression at follow-up and results are shown in Table 3. The odds of having symptoms of anxiety at follow-up were significantly increased for participants in the ADL category, and for depression in the MCI and ADL categories, relative to participants without cognitive impairment.

Logistic regression using the baseline data showed that the odds of having symptoms of anxiety or depression were higher in participants who reported SMC compared to those who did not report SMC (anxiety: OR = 3.05, CI = 1.79–5.20, *p* < .001; depression: OR = 2.01, CI = 1.57–2.58, *p* < .001).

A significant association was found between the presence of anxiety at baseline and the presence of SMC at follow-up (χ^2^ (1) = 12.56, *p* < .001). Based on the odds ratio, having anxiety at baseline increased the odds of having SMC at follow-up by a factor of 2.95. Partial correlation showed that the relationship between anxiety at baseline and SMC at follow-up remained significant when anxiety at follow-up was controlled for [*r* = .66, *p* (one-tailed) = .025] with anxiety at baseline accounting for 44% of the variance in SMC at follow-up. The association between the presence of depression at baseline and the presence of SMC at follow-up was also significant (χ^2^ (1) = 18.01, *p* < .001). Having depression at baseline increased the odds of having SMC at follow-up by a factor of 2.00 according to the odds ratio. Partial correlation showed that the relationship between depression at baseline and SMC at follow-up was significant when depression at follow-up was controlled for, with depression at baseline accounting for 41% of the variation in SMC at follow-up [*r* = .064, *p* (one tailed) = .027].

A significant association was found between the presence of anxiety at follow-up and the presence of SMC at follow-up (χ^2^ (1) = 37.62, *p* < .001). Based on the odds ratio, having anxiety at follow-up increased the odds of having SMC at follow-up by a factor of 8.75. The association between the presence of depression at follow-up and the presence of SMC at follow-up was also significant (χ^2^ (1) = 53.18, *p* < .001). This resulted in increased odds of having SMC at follow-up by a factor of 3.29 when depression was present at follow-up.

The presence of symptoms of anxiety or depression at baseline was not associated with progression from no cognitive impairment to MCI, OCIND, MCIW or dementia, or from MCI to dementia, over two years (see Table 4).

**Table 4.** Associations between symptoms of anxiety or depression and changes in cognitive status over two years.

		Pearson chi square	*p*
Anxiety at baseline			
Baseline	Follow-up		
NCI	MCI	0.14	.710
NCI	MCIW	0.52	.470
NCI	Dementia	0.37	.544
NCI	OCIND	0.56	.453
NCI	ADL	2.02	.155
MCI	Dementia	0.50	.480
MCIW	Dementia	0.36	.549
Depression at baseline			
Baseline	Follow-up		
NCI	MCI	0.02	.902
NCI	MCIW	0.27	.603
NCI	Dementia	0.01	.913
NCI	OCIND	0.17	.681
NCI	ADL	10.42	.001*
MCI	Dementia	0.38	.537
MCIW	Dementia	0.46	.499
Anxiety at follow-up			
Baseline	Follow-up		
NCI	MCI	0.19	.664
NCI	MCIW	2.12	.146
NCI	Dementia	0.56	.452
NCI	OCIND	0.49	.483
NCI	ADL	21.61	.000**
MCI	Dementia	0.04	.851
MCIW	Dementia	1.03	.310
Depression at follow-up			
Baseline	Follow-up		
NCI	MCI	9.88	.002*
NCI	MCIW	2.91	.088
NCI	Dementia	0.82	.366
NCI	OCIND	0.00	.984
NCI	ADL	18.98	.000**
MCI	Dementia	0.11	.743
MCIW	Dementia	2.06	.151

NCI = not cognitively impaired.

MCI = participants show objective cognitive impairment, intact general cognition, intact ADLs, no dementia, report of SMC.

MCIW represents participants who would otherwise be classified as MCI but do not report an SMC.

OCIND = other cognitive impairment, no dementia where participants indicate general cognitive decline but have intact ADLs and do not meet criteria for dementia.

ADL = participants who show general cognitive decline and impaired ADLs but do not meet criteria for dementia.

Dementia = participants who have been classified as having dementia.

*Significant at *p* = .05 level, **Significant at *p* < .001 level.

Symptoms of anxiety at follow-up were not associated with a change in cognitive status from no cognitive impairment to MCI, OCIND, MCIW or dementia, or from MCI to dementia, over two years. Symptoms of depression at follow-up were associated with a change in cognitive status from not cognitively impaired to a classification of MCI between baseline and follow-up (χ^2^ (1) = 9.72, *p* = .002) resulting in an increase in odds by a factor of four. However, symptoms of depression at follow-up were not associated with a change in cognitive status from no cognitive impairment to OCIND, MCIW or dementia, or from MCI to dementia, over two years (see Table 4). Depression at baseline and anxiety and depression at follow-up were associated with an increase in risk of developing ADL impairment in those with no cognitive impairment at baseline.

## Discussion

This study aimed to clarify the relationship between mood and SMC in people with MCI, in order to understand their appropriateness as a criterion in the MCI definition and add to the growing discussion around this. This study has used a novel approach to investigating the role of SMC by directly comparing their presence or absence in people who would otherwise meet criteria for MCI. The odds of having symptoms of anxiety or depression were increased in people with MCI compared to those without cognitive impairment or categorised as MCIW, but increases in odds were not seen for participants classified as MCIW. Logistic regression also indicated that the odds of reporting symptoms of anxiety or depression are increased in participants with SMC compared with participants without. Symptoms of anxiety and depression at baseline were significantly associated with the presence of SMC at follow-up but were not associated with a change in cognitive status between baseline and follow-up. Symptoms of depression at follow-up were significantly associated with a change in cognitive status from not cognitively impaired to a classification of MCI over two years, but symptoms of anxiety at follow-up did not show such an association.

The finding that people classified as having MCI have increased odds of experiencing symptoms of anxiety or depression compared to people without cognitive impairment is in line with the previous literature which has suggested that anxiety and depression are common comorbidities of MCI (Kruger et al., 2012; Ravaglia et al., 2008; Van der Linde, Stephan, Matthews, Brayne, & Savva, 2010). However, the odds of having symptoms of anxiety or depression are not increased in people classified as MCIW. This might suggest that the increase in odds for people with MCI is related to the SMC component of the MCI definition, as SMC are not a requirement for the MCIW category. Other research (Cook & Marsiske, 2006) suggests that depression does not drive the relationship between subjective beliefs and objective cognitive performance. However, no participants in the study by Cook and Marsiske (2006) endorsed depressive symptoms to a clinical level, whereas this study includes participants with clinical levels of depressive symptoms.

The odds of reporting symptoms of anxiety or depression are increased in participants reporting SMC compared to participants who did not report SMC. Again, this is in line with the previous literature (Balash et al., 2013; Caselli et al., 2013; Dux et al., 2008; Minett et al., 2008; Schmand, Jonker, Geerlings, & Lindeboom, 1997) and suggests that SMC and symptoms of anxiety or depression are related.

Data from two time points were used to investigate the relationship between SMC and symptoms of anxiety or depression over time, and participants who reported both anxiety and depression at baseline were more likely to have SMC two years later even after anxiety and depression at follow-up was controlled for. This suggests that anxious or depressive symptomology could influence how an individual appraises their memory or cognitive abilities, a possibility that has also been considered by other researchers (Dux et al., 2008; Jorm et al., 1997; Roberts et al., 2009).

Symptoms of anxiety and depression at baseline and anxiety at follow-up were not associated with changes in cognitive status between baseline and follow-up for either type of cognitive impairment (MCI or MCIW), with changes from normal cognitive functioning to dementia, or with changes from MCI to dementia. Symptoms of depression at follow-up were only associated with a change from not cognitively impaired to a classification of MCI. This contradicts previous research which has found that anxiety and depression are risk factors for cognitive decline (Caracciolo, Backman, Monastero, Winblad, & Fratiglioni, 2011; Geda et al., 2006; Goveas, Espeland, Woods, Wassertheil-Smoller, & Kotchen, 2011) but supports other research which has found that anxiety and depression may not be risk factors for progression from MCI to dementia (Gallagher et al., 2011; Vicini Chilovi et al., 2009).

Limitations of this study include the loss of participants at follow-up, leading to a relatively small number of participants reporting symptoms of anxiety at follow-up. Only data from participants who took part at both time points were used when assessing changes over the two-year period. Participants left the study between baseline and follow-up for several reasons, such as moving away from the study area, elective withdrawal and death.

Two years may not be enough time to track development of cognitive decline, SMC, or the development of symptoms of anxiety or depression. However, studies with longer follow-up periods have reported similar results (Comijs, Deeg, Dik, Twisk, & Jonker, 2002; Jorm et al., 1997).

Although the definitions of cognitive impairment used in this study rely on a participant's performance on an objective cognitive task, participants did not necessarily progress from no cognitive impairment to a type of cognitive impairment in a straightforward direction. The results show that 18.8% of participants classified as MCI and 21.6% of participants classified as having MCIW at baseline are classified as having normal cognitive functioning at follow-up, showing that many participants' cognitive performance had improved. This suggests that the categories of cognitive impairment used may lack stability. This result can also be found in other longitudinal studies (Palmer, Wang, Bäckman, Winblad, & Fratiglioni, 2002; Ritchie, Aterero, & Touchon, 2001) using a similar time frame to this study. The variable progression in MCI provides further evidence for the heterogeneous nature of MCI (DeCarli, 2003). Presence of SMC was also found to be unstable, with 38.1% of participants who had reported them at baseline no longer reporting them at follow-up.

Cognitive performance, SMC and symptoms of anxiety and depression were recorded as categorical data. A longer validated measure of SMC would have been preferable, and the items used did not cover all aspects. This does not allow for investigations into the variance that exists in cognitive performance and in mood-related experience and mediation or moderation analyses could not be performed.

Despite these possible limitations, this study has several strengths: the Cambridge Cognitive Examination (CAMCOG), is well established as a cognitive assessment tool for dementia and milder levels of cognitive impairment, and has been widely used in this area of research. The criteria used to create the MCI classification here are consistent with established definitions of MCI (Petersen, 2004; Petersen et al., 2001; Petersen et al., 1999). The procedure for assessment of mood-related symptoms used in this study has also previously been used to produce prevalence calculations for anxiety and depression which are in line with previous research and can be considered to be robust (Van der Linde et al., 2010). Lastly, this study uses a subsample drawn from a larger population sample, which is representative of the older population as participants were not identified through attendance at health services.

## Conclusions

This study has shown that people classified as having MCI are more likely to report symptoms of anxiety and depression people without cognitive impairment. Interestingly, participants in the MCIW group, who did not report SMC, were less likely than those without cognitive impairment to report anxiety and depression. People who reported SMC were more likely to also report mood problems regardless of their cognitive status. Participants who reported anxiety or depression at baseline were more likely to report SMC at the follow-up time point, but anxiety and depression at baseline were not associated with a change in cognitive status over two years. Only the presence of depression at the follow-up time point was associated with a change from not cognitively impaired to a classification of MCI.

The findings of this study also imply that a large number of participants who would otherwise meet criteria for MCI are missed, if SMC are seen as an essential criterion, and so may not receive appropriate support. Memory clinics can provide support, and access to interventions to improve memory and cognitive functioning, and can be provided upon referral from general practitioners. However, people who do not report memory problems are unlikely to seek such a referral, their objective cognitive problems are likely to remain unnoticed, and support that may benefit them in the long term is likely to remain inaccessible. More attention may also be needed for anxiety and depression in the context of MCI. This study suggests that SMC contribute significantly to the relationship between MCI and mood.
